# The Role of the Aryl Hydrocarbon Receptor (AHR) in Immune and Inflammatory Diseases

**DOI:** 10.3390/ijms19123851

**Published:** 2018-12-03

**Authors:** Drew R. Neavin, Duan Liu, Balmiki Ray, Richard M. Weinshilboum

**Affiliations:** 1 Mayo Clinic Graduate School of Biomedical Sciences, Department of Molecular Pharmacology and Experimental Therapeutics, Mayo Clinic, Rochester, MN 55902, USA; Neavin.Drew@mayo.edu; 2 Division of Clinical Pharmacology, Department of Molecular Pharmacology and Experimental Therapeutics, Mayo Clinic, Rochester, MN 55902, USA; Liu.Duan@mayo.edu (D.L.); rray@assurerxhealth.com (B.R.)

**Keywords:** aryl hydrocarbon receptor (AHR), single nucleotide polymorphisms (SNPs), tryptophan (TRP), aryl hydrocarbon response element (AHRE)

## Abstract

The aryl hydrocarbon receptor (AHR) is a nuclear receptor that modulates the response to environmental stimuli. It was recognized historically for its role in toxicology but, in recent decades, it has been increasingly recognized as an important modulator of disease—especially for its role in modulating immune and inflammatory responses. AHR has been implicated in many diseases that are driven by immune/inflammatory processes, including major depressive disorder, multiple sclerosis, rheumatoid arthritis, asthma, and allergic responses, among others. The mechanisms by which AHR has been suggested to impact immune/inflammatory diseases include targeted gene expression and altered immune differentiation. It has been suggested that single nucleotide polymorphisms (SNPs) that are near AHR-regulated genes may contribute to AHR-dependent disease mechanisms/pathways. Further, we have found that SNPs that are outside of nuclear receptor binding sites (i.e., outside of AHR response elements (AHREs)) may contribute to AHR-dependent gene regulation in a SNP- and ligand-dependent manner. This review will discuss the evidence and mechanisms of AHR contributions to immune/inflammatory diseases and will consider the possibility that SNPs that are outside of AHR binding sites might contribute to AHR ligand-dependent inter-individual variation in disease pathophysiology and response to pharmacotherapeutics.

## 1. Introduction

The aryl hydrocarbon receptor (AHR) is a ligand-activated transcription factor that was first identified as a result of its role in modulating the response to exogenous chemicals such as 2,3,7,8-tetrachlorodibenzo-p-dioxin (TCDD)—a contaminant of the chemical herbicide Agent Orange. However, in recent years, AHR has been appreciated as a crucial modulator of host-environment interactions [1,2,3,4,5], especially for immune and inflammatory responses [1,5,6,7,8,9,10,11]. The homeostasis of the immune and inflammatory systems has long been acknowledged as crucial for human health and disease prevention [3]. Elucidation of the role of AHR in disease and health is crucial to understand inter-individual variation in disease prevalence and therapeutic response and may aid in the development of novel therapies [12]. This review will focus on the role of AHR in modulating immune/inflammatory responses to pathogens, central nervous system diseases, the gut microbiome, inflammatory bowel disease, rheumatoid arthritis, and psoriasis. We will also discuss how common genetic polymorphisms may contribute to inter-individual variation in AHR-regulated disease prevalence and the AHR ligand-dependent response to therapeutic agents.

### 1.1. AHR Mechanism of Action

**Figure 1:**
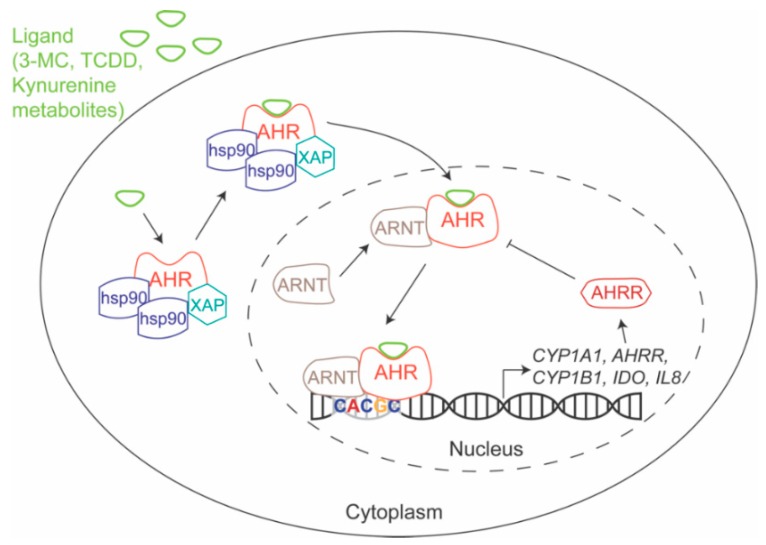
AHR Mechanism of Action. Prior to ligand binding, AHR is bound by cochaperones Hsp90 and XAP, which maintain its localization in the cytoplasm. After ligand binding, it is released from its cochaperones and is transported into the nucleus, where it heterodimerizes with ARNT and binds to DNA—often binding to AHREs (5′-CACGC-3′)—and regulates gene expression. *AHRR* is a prototypic AHR target gene and the encoded protein is a negative regulator of AHR. The arrows show the sequence of events that includes interaction, transport, DNA binding, gene expression and RNA translation. The T bar demonstrates that AHRR negatively regulates the AHR-ARNT interaction. AHR: aryl hydrocarbon receptor; AHRE: AHR response element; AHRR: AHR repressor; ARNT: aryl hydrocarbon receptor nuclear translocator; Hsp90: heat shock protein 90; XAP: X-associated protein 2.

AHR is a highly conserved nuclear receptor [6] that regulates gene expression after ligand binding. As a nuclear receptor, AHR is bound by cochaperones that maintain its localization in the cytoplasm. However, following ligand binding, AHR is released by the cochaperones and is transported into the nucleus, where it heterodimerizes with the aryl hydrocarbon receptor nuclear translocator (ARNT). The AHR-ARNT heterodimer regulates target gene expression by binding to DNA throughout the genome—often to AHR response elements (AHREs, 5′-GCGTG-3′) [13,14], which are also known as dioxin response elements (DREs) or xenobiotic response elements (XREs) [5,6]. AHR regulates many prototypic genes, including Cytochrome P450 Family 1 Subfamily A Member 1 (*CYP1A1*), Cytochrome P450 Family 1 Subfamily A Member 2 (*CYP1A2*), Cytochrome P450 Family 1 Subfamily B Member 1 (*CYP1B1*), TCDD Inducible Poly(ADP-Ribose) Polymerase (*TIPARP*), and aryl hydrocarbon receptor repressor (*AHRR*), which inhibits AHR through a negative feedback loop (Figure 1). Target gene regulation is thought to be, at least in part, ligand-dependent [14].

### 1.2. AHR Ligands

**Figure 2:**
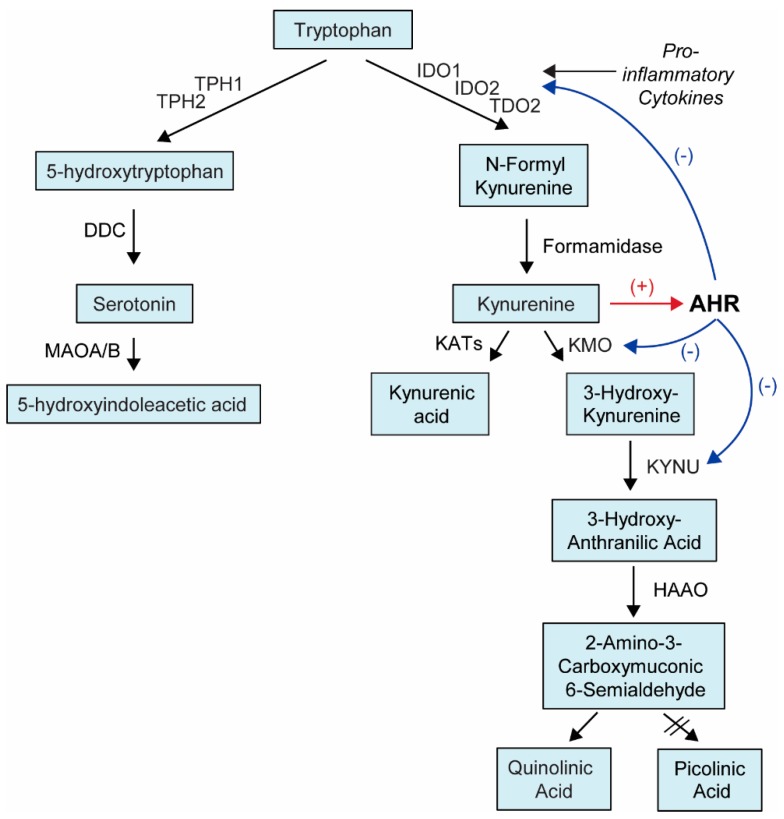
Tryptophan Metabolism Pathway. Tryptophan is metabolized by two main pathways: the serotonin pathway (~1% of tryptophan metabolism) and the kynurenine pathway (~95% of tryptophan metabolism). The rate limiting enzymes IDO1/2 can be induced with pro-inflammatory cytokines, resulting in the production of more kynurenine, which is an agonist for AHR. AHR can also regulate the expression of IDO1/2, TDO2, KYNU, and KMO. The black arrows indicate enzymatic reactions, the red arrows indicate positive (+) regulation and the blue arrows indicate negative (−) regulation. DDC: dopa decarboxylase; HAAO: 3-hydroxyanthranilate 3,4-dioxygenase; IDO: indoleamine 2,3-dioxygenase; KATs: kynurenine amino transferases; KMO: Kynurenine 3-Monooxygenase; KYNU: Kynureninase; MAOA/B: monoamine oxidase A/B; TDO2: tryptophan 2,3-dioxygenase; TPH1/2: tryptophan hydroxylase.

AHR is a highly promiscuous nuclear receptor that can bind many diverse ligands. Those ligands include exogenous synthetic aromatic hydrocarbons [6,15], exogenous natural chemicals [1,3,6,10,16], and endogenous ligands [1,3,5,7,8,10,17,18,19,20,21]. Examples of common AHR ligands are listed in Table 1. Specifically, the tryptophan (TRP) pathway provides many ligands for AHR and plays an important role in immune and inflammatory responses. Further, AHR regulates the expression and activation of indoleamine 2,3-dioxygenase (IDO), tryptophan 2,3-dioxygenase (TDO2), kynureninase (KYNU), and kynurenine 3-monooxygenase (KMO), which are enzymes that regulate the kynurenine (KYN) arm of TRP metabolism, thereby providing a feedback loop since KYN is an agonist for AHR (Figure 2) [22,23].

## 2. AHR-Dependent Pathogen Response

### 2.1. Microbial Pathogens

AHR has been shown to play an important role in modulating the response to many microbial pathogens. In this context, lipopolysaccharide (LPS) stimulation has frequently been used as a model for gram-negative sepsis to study the role of AHR in infection control and the modulation of septic shock. AHR and TDO2 are required for survival after the initial exposure to LPS [15,21], while subsequent exposures are dependent on AHR and IDO1/2. LPS stimulates the increased expression of TDO2 and IDO1/2, which are the rate limiting enzymes for TRP metabolism to KYN (Figure 2), which then activates AHR, which decreases the expression of pro-inflammatory cytokines and regulates long-term systemic inflammation [21]. Further, AHR^−/−^ mice or immune cells that are challenged with LPS produce higher concentrations of the pro-inflammatory cytokines interleukin 1 beta (IL-1β), IL-18, interferon gamma (IFN-γ), tumor necrosis factor alpha (TNF-α), IL-12, and IL-6, as well as NLR Family Pyrin Domain Containing 3 (NLRP3), which regulates the expression of many pro-inflammatory cytokines compared to AHR wildtype (WT) mice or immune cells [15,32,33,34,35,36,37,38]. Further, the AHR agonists 3-Methylcholanthrene (3-MC), TCDD, 6-Formylindolo[3,2-b]carbazole (FICZ), and KYN can protect AHR WT, but not AHR^−/−^, mice from excessive pro-inflammatory cytokine expression and septic shock [15,38].

An effective response to the gram-positive pathogen *Listeria Monocytogenes* (LM) also requires AHR. In a murine model, AHR was shown to protect against LM by promoting the formation of reactive oxygen species (ROS), by increasing the expression of the anti-inflammatory cytokine IL-10 and apoptotic inhibitor of macrophages, which results in decreased macrophage apoptosis, as well as decreased expression of pro-inflammatory cytokines (IL-6 and TNF-α) and decreased activation of the nuclear factor kappa-light-chain-enhancer of activated B cells (NF-κB). Further, similar to the gram-negative murine model, AHR ligands were able to enhance the response to LM in AHR WT mice, but not AHR^−/−^ mice [19]. Therefore, AHR plays an important role in the response to both gram-negative and gram-positive pathogens and understanding these mechanisms may make it possible to identify novel therapeutic agents that can be used to combat microbial pathogens.

### 2.2. Viral Pathogens

AHR has also been implicated in the viral pathogenic response. Specifically, ocular infection from herpes simplex virus can result in a chronic immune-inflammatory reaction that can result in blindness, but a single dose of TCDD in a murine model was able to relieve herpes keratitis lesions, decrease the viral load, and decrease pro-inflammatory cytokines. However, FICZ did not demonstrate the same efficacy, thus illustrating differences between these two AHR ligands [39]. Therefore, AHR is required for the response to at least one viral pathogen and a non-toxic AHR agonist may have the capability to treat an ocular infection from the herpes simplex virus.

### 2.3. Parasitic Pathogens

AHR is also important for the response to parasitic pathogens. *Toxoplasma gondii* is a parasitic pathogen that causes toxoplasmosis. The response to *Toxoplasma gondii* requires AHR-dependent increased expression of the anti-inflammatory cytokine IL-10. An AHR^−/−^ murine model demonstrated a decreased response to *Toxoplasma gondii* and a smaller increase in IL-10 [40]. Therefore, AHR is required for at least one parasitic pathogen and provides information about one response pathway that could be used to design novel therapies.

## 3. AHR and the Central Nervous System

### 3.1. AHR and Major Depressive Disorder

We have recently demonstrated that AHR is associated with inter-individual variation in the plasma KYN concentration in major depressive disorder (MDD) patients and that variation in the KYN concentration was also associated with MDD severity. Further, we demonstrated that AHR regulated the expression of the rate-limiting KYN pathway enzymes TDO2 and IDO1/2, as well as the downstream enzymes kynureninase (KYNU) and kynurenine 3-monooxygenase (KMO). In vitro cell culture experiments that modulated AHR activity demonstrated that AHR knock down (KD) resulted in a decreased KYN concentration in cell culture media, likely due to the increased downstream metabolism of KYN that produces quinolinic acid, which is a neurotoxic NMDA receptor agonist that can contribute to MDD symptoms [23].

### 3.2. AHR and Multiple Sclerosis

Multiple sclerosis (MS) is an immune-mediated demyelinating disease [41]. Patients with MS have lower levels of circulating AHR than healthy controls, which indicates that AHR may play a role in MS pathogenesis [42]. In an MS model, experimental autoimmune encephalitis (EAE) [6], AHR KD increased disease scores, while the activation of AHR with AHR agonists, such as TCDD, indole-3-carbinol (I3C), and diindolylmethane (DIM), suppressed EAE disease progression through increased Forkhead Box P3 (FOXP3) expression, increased anti-inflammatory regulatory T cells (Treg), and decreased pro-inflammatory Th17 expansion [6,14,18,42]. FICZ, another AHR agonist, also alleviated EAE disease progression and decreased EAE disease scores in murine models when administered systemically [43]. However, the local administration of FICZ increased Th17 expansion, which resulted in increased EAE disease scores [6,18]. The mechanism by which FICZ results in different AHR activation responses is not fully understood, but is linked to opposing effects on Th17 cell populations, the administration method, and the dose. Further, AHR plays an important role in the contribution of the gut-brain axis to EAE disease severity by regulating astrocyte inflammation and EAE disease scores. The removal of dietary TRP increases EAE disease scores, but EAE disease scores can be decreased after the reintroduction of TRP in AHR WT mice, but not in AHR^−/−^ mice [42]. Finally, laquinimod is a drug currently under development that passes the blood brain barrier (BBB) and ameliorates EAE in an AHR-dependent manner [8]. Therefore, AHR may be an important modulator of MS and could be a therapeutic target for future MS drug development.

### 3.3. AHR and Congenital Nystagmus

Congenital nystagmus is a condition that results in involuntary eye movements and is associated with many central nervous system (CNS) pathologies [44]. AHR^−/−^ mice demonstrated congenital nystagmus, which suggests that AHR may play an important role in the pathogenesis of congenital nystagmus and related CNS pathologies [44]. AHR^−/−^ mice demonstrated the dysregulation of myelin structure, increased pro-inflammatory cytokine gene expression, and STAT1 target gene dysregulation, all of which may contribute to congenital nystagmus [44].

Therefore, AHR appears to play crucial roles in multiple CNS diseases, including MDD, MS, and congenital nystagmus, and it is also important for the gut-brain axis, which may contribute to these and other CNS diseases.

## 4. AHR and the Gut Microbiome

AHR is highly expressed in epithelial barriers [17,45] and AHR^−/−^ mice have an insufficient gut barrier, which indicates that AHR may be important for either sustaining or developing healthy gut barriers [20]. Further, AHR expression and the expression of AHR target genes are lower in germ-free mice [10] and AHR is required to maintain a balance of RORγt^+^ innate lymphoblastoid cells (ILCs) in the gut [18]. Further, TRP metabolite indoles produced by some gut microbiome bacteria are ligands for AHR [46,47] and a diet without indoles or treatment with antibiotics results in decreased AHR-dependent mononuclear phagocyte differentiation into dendritic cells (DCs) [47] and higher susceptibility to intestinal pathogens in murine models [18]. Collectively, these data indicate that AHR may be important for the host gut-microbiome interaction.

## 5. AHR and Inflammatory Bowel Disease

Ulcerative colitis (UC) and Crohn’s Disease (CD) are the two main types of inflammatory bowel disease (IBD), which are autoimmune inflammatory diseases. Expression levels of AHR are lower in CD but not UC patients and AHR expression is especially decreased in the inflamed mucosa of CD patients [48]. In addition, decreased levels of endogenous AHR ligands have been reported in IBD patients compared with healthy controls [42]. Therefore, AHR activity may be related to IBD symptoms. In murine models, dextran sulfate sodium (DSS)-induced colitis demonstrated more severe symptoms and higher levels of pro-inflammatory cytokines in AHR^−/−^ mice than WT controls [49]. Further, the AHR ligands TCDD, Norisoboldine (NOR), and FICZ can relieve colitis symptoms by acting through AHR [18,50,51]. TCDD ameliorated colitis symptoms by suppressing Th17 cell differentiation, resulting in decreased IL-17 and IFN-γ expression, while NOR reduced colitis by promoting Treg differentiation and inhibiting the NLRP3 inflammasome [50,51]. FICZ protected against the development of colitis symptoms by decreasing pro-inflammatory cytokine production (IL-17, IL-1β, IL-6, TNF-α, and IFN-γ) and by increasing anti-inflammatory IL-22 production from Th17 cells [18,48,49]. Therefore, AHR may be an important mediator of IBD and natural diet-consumed AHR ligands may be potential therapeutic targets for IBD and UC.

## 6. AHR and Rheumatoid Arthritis

Rheumatoid arthritis (RA) is a chronic inflammatory disease that presents with synovial inflammation and bone and cartilage erosion [52] that affects ~1% of the population [3]. This complex disease is influenced by both environmental and genetic factors [11]. Environmental pollutants and cigarette smoke are associated with RA disease risk—both of which contain agonists for AHR [11].

Murine models of RA have demonstrated that AHR activation with TCDD or FICZ can contribute to RA disease progression, disease severity, bone destruction, osteoclasts differentiation, and increased numbers of IL17-expressing cells in the inflamed joints [11]. Further, AHR^−/−^ mice have demonstrated decreased serum concentrations of the pro-inflammatory cytokines matrix metalloproteinase 3 (MMP-3), IL-1β, and IL-6, resulting in decreased disease severity, which is largely T cell-dependent and not macrophage-dependent [3]. Further, the role of AHR in RA symptoms is modulated by NF-κB. However, paradoxically, NOR, a chemical from Radix Linderae that acts as an AHR agonist, has been shown to decrease RA severity in rats by decreasing osteoclast differentiation [52].

Clinical samples have also demonstrated that AHR expression is about two-fold higher in RA patients than in controls [9] and that CYP1A1 and AHRR expression are increased in the synovia of RA patients who smoke cigarettes, but not in the synovia of patients who do not smoke, which indicates that there is a potential interaction between cigarette smoke and AHR activation in RA patients [53]. Collectively, these data suggest a role for AHR in modulating the response to the environment that may contribute to RA disease severity.

## 7. AHR and Psoriasis

Psoriasis vulgaris is a complex chronic autoimmune disorder with environmental and genetic components that affects 2–3% of the American population [54,55]. Many genes that are dysregulated during psoriasis are increased after AHR antagonist exposure or in AHR^−/−^ murine models, which results in excessive inflammatory response [7,20]. Further, treatment of an imiquimod-induced psoriasis murine model with the AHR agonist FICZ decreased psoriasis and decreased the expression of psoriasis-related genes, effects which were dependent on the stromal expression of AHR and interaction with the adaptive immune system [7,20].

Tapinarof, a naturally-derived topical treatment, which is in phase III clinical trials for the treatment of psoriasis vulgaris, has been demonstrated to act as an AHR agonist. Tapinarof decreases imiquimod-induced lesions in murine models and decreases the expression of the pro-inflammatory cytokines IL-17A and IL-22. However, while tapinarof clearly has anti-inflammatory properties, it is not preventative [54]. Therefore, AHR may be a regulator of psoriasis and other chronic inflammatory skin diseases.

## 8. AHR and Atherosclerosis

Atherosclerosis is a chronic inflammatory disease of the arteries [56] that is influenced by both genetic and environmental components. Tobacco and cigarette smoke, which contain many AHR ligands [57,58,59], are associated with an increased risk of atherosclerosis [56,59,60,61]. Further, a single nucleotide polymorphism (SNP) that is 5′ of the *AHR* gene and is associated with variation in AHR expression has been associated with atherosclerosis [62]. Murine models exposed to AHR ligands such as BaP (an environmental contaminant found in tobacco smoke) or TCDD resulted in increased atherosclerosis. In a murine model that compared high and low affinity AHR, atherosclerosis further increased with low affinity AHR [56,57,59]. In addition, AHR is highly expressed in the atherosclerotic portions of arteries in both mouse models and human samples [62]. AHR ligand-induced atherosclerosis has been linked to altered AHR-regulated redox-pro-inflammatory events and interactions with the transcription factor TCF21, which plays an important role in atherosclerosis [61,62]. These results indicate that AHR activity may modulate the effect of toxic AHR ligands, including tobacco smoke, on atherosclerosis risk. Therefore, it has been suggested that AHR antagonists may be a potential therapeutic option for the treatment and/or prevention of atherosclerosis [61].

## 9. AHR and Single Nucleotide Polymorphisms

SNPs are common, single nucleotide genetic variants that can influence protein function, protein stability, or gene expression. Expression quantitative trait loci (eQTLs)—SNPs that are associated with variation in gene expression—have been identified for AHR and have been associated with important phenotypes, such as MDD [23], atherosclerosis [62], and coffee consumption [63]. Those findings implicate AHR as an important contributor to MDD, atherosclerosis, and coffee consumption.

Genome-wide association studies and candidate gene studies have identified SNPs near AHR target genes that are significantly associated with AHR-regulated phenotypes, such as cigarette smoking (*AHRR*) [64], psoriasis (*CYP1A1*) [17], warfarin response (*CYP1A1*) [65], hepatocellular carcinoma (*CYP1A1*) [66], atherosclerosis (*CYP1A1* and *AHRR*) [57,58,67,68,69], coffee consumption (*CYP1A1* and *CYP1A2*) [63], and systemic lupus erythematous (*CYP1A1*) [17]. These findings suggest that SNPs near AHR binding sites might impact AHR target gene expression and contribute to individual variation in disease risk and pharmacotherapy phenotypes [17].

Further, previous studies have identified an SNP in the promoter of *CYP1A1* that was only an eQTL after AHR agonist pharmacological treatment—termed a pharmacogenomic (PGx)-eQTL [70]. Of interest, that SNP is distant from an AHR response element (AHRE), but still influences AHR binding and CYP1A1 expression after AHR agonist treatment, which suggests that it may influence the stability of the AHR complex and its ability to regulate *CYP1A1* gene expression [70]. Previous findings of other PGx-eQTLs for another nuclear receptor, estrogen receptor alpha (ERα) [70,71,72,73,74,75], indicate that this may be a general phenomenon and that additional SNPs distant from AHR binding sites may function as PGx-eQTLs by impacting ligand-dependent gene expression.

## 10. Discussion

We have presented examples of diseases in which AHR modulates disease by interaction with environmental stimuli and outlined the currently understood molecular mechanisms that contribute to those disease phenotypes. Clearly, the role of AHR is model-specific, disease phenotype-specific, and ligand-specific [18], with AHR activation contributing to the symptoms of some diseases, but relieving the symptoms of other diseases (Table 2). Further, in some cases (i.e., RA and MS), the result and effect of AHR activation is dependent on the ligand that is studied [3,6,11,14,18,42,52].

Natural, AHR ligands, including AHR agonists, and selective AHR modulators (SARMs), have been suggested as potentially safe and effective therapeutic treatments that may be useful for the treatment of immune and inflammatory disease phenotypes that are regulated by AHR. However, it is crucial to first understand the exact molecular pathways that are impacted by each ligand across different tissues due to the possible toxic, carcinogenic, and teratogenic effects of AHR ligands [1,4,6,8,15,17,18,19,20,32,33,34,35,36,38,39,46,47,76,77,78,79]. Advances in nanotechnology may provide novel delivery methods that would permit highly tissue-specific targeting of AHR ligands that could avoid systemic AHR ligand exposure [14]. In fact, some AHR agonists have already been shown to be effective and safe treatments for specific disease phenotypes (Table 3) [10,16,18,54]. Further, some pharmacotherapeutic agents that have been used and determined to be safe by the US Food and Drug Administration, such as leflunomide, sertraline, prednisolone, and omeprazole, were recently shown to act as AHR agonists [54].

Finally, SNPs that are near AHR binding sites have been shown to alter AHR ligand-dependent target gene expression [70]. Unpublished work from our laboratory indicates that this is not a singular event and that there may be many SNPs throughout the genome that are distant from AHR binding sites that impact AHR ligand-dependent gene expression. Therefore, SNPs throughout the genome that impact AHR ligand-dependent gene expression may contribute to inter-individual variation in disease prevalence, disease severity, and pharmacotherapeutic response.

Therefore, AHR is an important environmental modulator that contributes to the prevalence and severity of many immune and inflammatory diseases and which has the potential to serve as a target for novel pharmacotherapeutic agents. However, more research is needed with regard to the long-term effect of those agents and they should be considered for possible use in the clinic, but only with extreme caution due to the broad role of AHR in immune regulation and the toxic effect of some AHR ligands.

## Figures and Tables

**Table 1:** AHR ligands. A list of common AHR ligands, their abbreviations, their EC_50_ values, whether they are exogenous or endogenous, whether they are synthetic products or natural products, and their source. The table is sorted by EC_50_ value.

Ligand	Abbreviation	EC_50_ (M) *	Exogenous/Endogenous	Synthetic/Natural	Source
2,3,7,8-Tetrachlorodibenzo-p-dioxin	TCDD	10^−11^–10^−9^ [24,25,26,27,28]	Exogenous	Synthetic	Chemical contaminant (i.e., Agent orange contaminant)
6-Formylindolo[3,2-b]carbazole	FICZ	10^−11^–10^−10^ [29,30]	Endogenous	Natural	Ultraviolet derivative of tryptophan
Benzo[a]pyrene	BaP	10^−9^–10^−8^ [28,31]	Exogenous	Synthetic	Product of burning of organic compounds and cigarette smoke
3-Methylcholanthrene	3-MC	10^−9^–10^−6^ [27,28,30]	Exogenous	Synthetic	Product of burning of organic compounds
Kynurenine	KYN	10^−9^–10^−5^ [29]	Endogenous	Natural	Tryptophan metabolite
β-napthoflavone	BNF	10^−8^ [31]	Exogenous	Synthetic	Flavone derivative
α-napthoflavone	ANF	10^−7^ [28]	Exogenous	Synthetic	Flavone derivative
Indolo[3,2-b]carbazole	ICZ	10^−7^ [25]	Endogenous	Natural	Indole-3-carbinol derivative
Diindolylmethane	DIM	10^−5^ [30]	Exogenous	Natural	Indole-3-carbinol derivative
Indole-3-carbinol	I3C	10^−3^–10^−5^ [31]	Exogenous	Natural	Cruciferous vegetables
Tryptophan	TRP	10^−4^ [31]	Exogenous	Natural	Essential amino acid
Indole-3-acetic acid	IAA	10^−4^ [26,31]	Exogenous & Endogenous	Natural	Microbiome product Tryptophan metabolite
Tryptamine	TRYP	10^−4^ [26,31]	Endogenous	Natural	Tryptophan metabolite
Norisoboldine	NOR	NA	Exogenous	Natural	Alkaloid isolated from Radix Linderae

* Note: Methodologies for obtaining EC_50_ concentrations vary between publications, which can impact results. Readers are urged to refer to the original articles.

**Table 2:** Effect of AHR on disease phenotypes and pathways implicated in the effect.

Disease	AHR Ligands	AHR Activation Phenotype	AHR Inactivation Method	AHR Inactivation Phenotype	Contributing Pathways	References
LPS-induced septic shock	3-MC, TCDD, FICZ, KYN	Decreased death	AHR^−/−^	Increased death	IDO/TDO activation; TRP metabolism	[15,21,38]
*Listeria Monocytogenes*	TCCD, FICZ	Decreased death	AHR^−/−^	Increased death	ROS formation and cytokine expression	[19]
Herpes-simplex virus-induced ocular Infection	TCDD	Decrease herpes keratitis lesions	NA	NA	Unclear but decreased numbers of inflammatory IFN-γ+ secreting CD4+ T cells (Th1) and Th17 cells	[39]
	FICZ	No effect				
*Toxoplasma gondii* infection	NA	NA	AHR^−/−^	Decreased anti-inflammatory response	IL-10 expression	[40]
Major depressive disorder	AHR SNP eQTL, 3-MC	Worse MDD symptoms, increased KYN	AHR SNP eQTL, AHR KD	Less MDD symptoms, decreased KYN	TRP metabolism; IDO/TDO, KMO, KYNU activation	[23]
Multiple Sclerosis	TCDD, I3C, DIM	Decreased disease scores	AHR KD	Increased disease scores	FOXP3 expression, Treg expansion, Th17 expansion	[6,8,14,18,41,42,43]
	FICZ	Systemic exposure: decreased disease scores; Local administration: increased disease scores				
Congenital nystagmus	NA	NA	AHR^−/−^	Development of congenital nystagmus	Proinflammatory cytokine expression, STAT1	[44]
Gut microbiome	TRP indoles	DC differentiation, ILC balance	AHR^−/−^, Remove diet TRP, antibiotics	Pathogen susceptibility, ILC balance, DC differentiation	Unclear	[17,18,45,46,47]
Inflammatory Bowel Disease	TCDD, NOR, FICZ	Relieve colitis symptoms	AHR^−/−^	More severe colitis symptoms	Pro-inflammatory cytokine expression, Th17 differentiation, Treg differentiation, NLRP3 inflammasome expression	[18,48,49,50,51]
Rheumatoid Arthritis	TCDD, FICZ	Increased disease severity	AHR^−/−^	Decreased disease severity	Pro-inflammatory cytokine expression, NF-κB	[3,9,11,43,52,53]
	NOR	Decreased disease severity				
Psoriasis	FICZ, tapinarof	Decreased disease severity	AHR^−/−^	Increased disease severity	Pro-inflammatory cytokine expression, keratinocyte interaction with adaptive immune system	[7,20,54,55]
Atherosclerosis	TCDD, BaP	Increased disease severity	AHR low affinity	Increased disease severity	Pro-inflammatory cytokine expression, reactive oxygen species, TCF21 interactions	[56,57,58,59,60,61,62]

**Table 3:** AHR ligands with potential clinical utility.

Compound	Administration Method	Target Diseases	Comments
Tapinarof	Topical	Psoriasis	Currently in Phase III clinical trials; highly effective; safe
NOR	Depends	IBD, RA	Clinical safety studies will be required
I3C/DIM	Oral	IBD, MS, gut microbiome balance	Normally consumed in cruciferous vegetables

## Abbreviations

3-MC	3-methylcholanthrene
AHR	aryl hydrocarbon receptor
AHRE	aryl hydrocarbon receptor response elements
AHRR	aryl hydrocarbon receptor repressor
ARNT	aryl hydrocarbon nuclear translocator
ANF	α-napthoflavone
BNF	β-napthoflavone
CD	Crohn’s disease
CNS	central nervous system
COPD	chronic obstructive pulmonary disease
CYP1A1	cytochrome P450 Family 1 Subfamily A Member 1
CYP1A2	cytochrome P450 Family 1 Subfamily A Member 2
CYP1B1	cytochrome P450 Family 1 Subfamily B Member 1
DC	dendritic cell
DIM	diindolylmethane
DSS	dextran sulfate sodium
EAE	experimental autoimmune encephalitis
eQTL	expression quantitative trait locus
FICZ	6-formylindolo[3,2-b]carbazole
FOXP3	forkhead Box P3
I3C	indole-3-carbinol
IAA	indole-3-acetic acid
IBD	inflammatory bowel disease
ICZ	indolo[3,2-b]carbazole
IDO	indoleamine 2,3-dioxygenase
IFN-g	interferon gamma
IL-12	interleukin 12
IL-17	interleukin 17
IL-18	interleukin 18
IL-1β	interleukin 1 beta
IL-22	interleukin 22
IL-6	interleukin 6
ILC	innate lymphoblastoid cell
KD	knock down
KMO	kynurenine 3-monooxygenase
KYN	kynurenine
KYNU	kynureninase
LM	*Listeria Monocytogenes*
LPS	lipopolysaccharide
MDD	major depressive disorder
MS	multiple sclerosis
NF-kB	nuclear factor kappa-light-chain-enhancer of activated B cells
NLRP3	NLR Family Pyrin Domain Containing 3
NOR	Norisoboldine
PGx	pharmacogenomic
RA	rheumatoid arthritis
ROS	reactive oxygen species
SNP	single nucleotide polymorphism
TCDD	2,3,7,8-tetraclorodibenzo-p-dioxina
TDO2	tryptophan 2,3-dioxygenase
TIPARP	TCDD Inducible poly(ADP-Ribose) polymerase
TNF-a	tumor necrosis factor alpha
UC	ulcerative colitis
WT	wildtype
